# Prevalence, risk factors and multiple outcomes of treatment delay in Chinese patients with schizophrenia

**DOI:** 10.1186/s12888-023-05247-0

**Published:** 2023-10-13

**Authors:** Yue-Hui Yu, Quan Lu

**Affiliations:** 1 School of Public Administration and Policy, Renmin University of China, Beijing, 100872 China; 2 School of Labor and Human Resources, Renmin University of China, Beijing, 100872 China

**Keywords:** Schizophrenia, Treatment delay, Risk factors, Outcomes, China

## Abstract

**Background:**

People with schizophrenia often delay treatment. This issue is not fully understood, particularly in low-and middle-income countries. This study aimed to elucidate the prevalence, risk factors and multiple outcomes of treatment delay in schizophrenia in a Chinese metropolis.

**Methods:**

A two-stage whole cluster sampling survey was conducted in Beijing, China in 2020. A total of 1,619 patients with schizophrenia were included. Heterogeneity between groups and the changing trend of treatment delay were presented. Regression modelling methods were used to examine both the risk factors for treatment delay and related outcomes at individual and family levels.

**Results:**

The median treatment delay for schizophrenia was 89 days (about 13 weeks). 49.35% surveyed patients delayed treatment for more than three months. Early age of onset, low level of education, living in well developed districts were important risk factors. Treatment delay in schizophrenia was significantly associated with patients’ poor medication adherence, comorbidity status and poor social functioning. It also increased the negative impact of the illness on families.

**Conclusions:**

This study accumulated evidence of treatment delay in schizophrenia in China. It occurs even in the metropolis where mental health resources are relatively adequate. Further targeted interventions to raise public awareness should be crucial to reduce treatment delay.

## Introduction

Schizophrenia is a severe mental illness that affects around 24 million people worldwide, or 1 in 300 people (0.32%) [1]. It is associated with high levels of comorbid psychopathology, premature mortality, and significant socioeconomic burden [2, 3]. It appears that the first few years after disease onset are more variable compared to the later years, when symptom levels and functional deficits seem to stabilize [4, 5]. Therefore, early treatment and intervention are important to reduce the burden of disease.

However, people with schizophrenia often delay accessing mental health services, the reasons for which are not yet fully understood [6, 7]. The interval between the onset of psychosis and the first psychopharmacological treatment, commonly referred to as the ‘the duration of untreated psychosis’, is alarmingly long [8, 9]. Treatment delays in schizophrenia are associated with factors such as nationality, ethnicity, age of disease onset, marital status, educational attainment, income status, and many others [10, 11].

Treatment delay may be highly contextualized and culturally relevant [12]. Current evidence is inconsistent regarding the ways in which factors may influence treatment delay in schizophrenia. For example, older age at onset and being single are associated with longer treatment delay in some studies [13, 14], whereas they are protective factors in others [15, 16]. Similar inconsistencies also exist in the interpretation of its outcomes. Although its physiological effects are widely recognized, the extent and nature of the effects of delayed treatment vary across studies rooted in different contexts [15, 17, 18].

In China, the lifetime prevalence of schizophrenia is 0.6% [19], with approximately 7 million patients are affected by schizophrenia. Providing timely treatment to minimize the disease burden has become a major public health challenge. Studies demonstrating the prevalence, risk factors, and potential outcomes of treatment delay in schizophrenia in the Chinese context are still rare [20–22]. Using cross-sectional data collected from a Chinese metropolis, this study aimed to reveal the status quo of treatment delay in schizophrenia in China, identify its risk factors and examine its clinical and social outcomes.

## Methods

### Data source

The data in this study is derived from the Survey on the Economic Burden and Free Medication Services of Mental Illness in Beijing, conducted between July and December, 2020. The purpose of this survey was to investigate the living and treatment status of people with severe mental illness. It was conducted using a whole cluster sample, which was completed in two stages. The research group first sampled community or township health centers, two randomly selected from each of the 14 districts and one randomly selected from each of the two districts with a smaller population. This resulted in a total of 30 health centers randomly selected from all 16 districts to form the overall sample frame. The selected health centers then provided the full list of people with severe mental illness. Trained interviewers approached the patients and their caregivers. They asked them a series of questions and immediately recorded the answers. A total of 2,994 people with severe mental illness were surveyed. Among then, only those with schizophrenia were taken into consideration in this study (N = 1619). This study was approved by the Research Ethics Committee of the School of Public Administration and Policy, Renmin University of China (EA-NSFC72204256).

### Variable measurements

#### Treatment delay

Treatment delay was defined as the length of time between the first onset of schizophrenia and the start of psychiatric treatment. In this study, the variable ‘treatment delay’ was downgraded to an ordered variable because it did not have a normal distribution even after transformation. It was divided into three categories: no delay (days of delay ≤ 90), moderate delay (90 < days of delay ≤ 365*2) and severe delay (days of delay > 365*2). This division is based on existing clinical research [23], which suggests that the first three months of disease onset is the acute phase, when treatment is most effective. If treatment is delayed for more than two years, the prognosis can be very poor. Treatment delay was used a dependent variable to explore its risk factors, and as an independent variable to explore its multiple outcomes.

#### Outcome measurements

We measured the outcomes of treatment delay at both the individual and household levels. At the individual level, we focused on the clinical outcomes, operationalized by medication adherence (yes/no), comorbidity status (yes/no) and a simplified assessment of social functioning (poor/good). To monitor the treatment and recovery process of severe mental illness, psychiatrists in China are responsible for assessing patients’ social functioning according to criteria set by hospitals. The initial assessment of social functioning is divided into five levels (i.e. extremely poor, poor, fair, good, very good). In this study, those rated as having good or very good social functioning represented 74.68% of the total sample and we reclassified them as ‘good’, while the others were reclassified as having poor social functioning.

At the household level, we tested the impact of the disease as perceived by caregivers by asking them to rate items such as ‘income of other family members has been affected’, ‘normal family leisure activities have been affected’. A total of 24 items were rated on a three-point Likert scale, anchored at 1 = no impact, 2 = moderate impact and 3 = strong impact. These 24 items were grouped into four subscales measuring four variables, including the economic burden (6 items), daily life burden (9 items), relationship burden (5 items) and health burden (4 items). The scores on each subscale were summed to give an overall score for each variable, with higher overall scores indicating more negative outcomes.

#### Control variables

Control variables included gender, age, age at onset, marital status (married/unmarried), educational level (primary school or below, junior high school, high school and above), residential status (rural/urban), family history of mental illness traced in blood relatives over three generations (yes/no), highest level of family member’s education (above/below college level), family economic status (poor/not poor), and the level of development of the residential areas as measure by GDP (high, medium, relatively low).

### Statistical analysis

Stata 15.0 was used for data analysis. To ensure a high completion rate, the questionnaire was administered and completed by trained interviewers rather than by the patients themselves. For certain questions that the patients could not answer, their caregivers provided answers. The quality control group also monitored the data collection process to minimize missing questions. In the original data there were 55 cases with missing values and we dropped these cases. Data analysis was carried out in four steps. First, descriptive analysis of the distribution of variables and Pearson chi-square results were reported for the study population (n = 1,619). Treatment delay was also presented in figures to show its trend over time. Second, ordered logistic regressions were used to examine the risk factors for treatment delay, with the base being the ‘no delay’ group. Third, binary logistic regressions were performed to estimate the clinical outcomes related to treatment delay on medical adherence, comorbidity status and social function of patients. Finally, linear regression models were used to examine household level outcomes of schizophrenia, with treatment delay and its clinical outcomes all as independent variables. Odd ratio (OR), unstandardized coefficient (B), standard error (St.Err.) and significance level are reported where appropriate.

## Results

### Social and clinical characteristics

Table 1 shows the social and clinical characteristics of the participants and the test scores for treatment delay status in each category (n = 1,619). The duration of treatment delay varied from 0 to 23,701 days (mean 1,557.66; median 89). Participants who received treatment within 3 months of onset (no delay group) accounted for 50.65% of the total sample. The moderate and severe delay groups accounted for 20.63% and 28.72%, respectively. They were aged between 18 and 94 years (mean 54.44; median 55) and their age at onset varied from 13 to 84 years (mean 30.14; median 28).

**Table 1:** Social and clinical characteristics of participants (N = 1,619)

Characteristics	Mean	SD	Median	Range	
Treatment delay (days)	1,557.66	3406.79	89		0–23,701
Age	54.44	13.43	55		18–94
Age at onset	30.14	12.67	28		13–84
			**Test by treatment delay status (three categories)**		
	**N**	**%**	**Chi-square**		**p**
**Treatment delay status (three categories)**					
No delay	820	50.65			
Moderate delay	334	20.63			
Severe delay	465	28.72			
**Gender**			4.165		0.125
Male	699	43.17			
Female	920	56.83			
**Marital status**			13.692		0.001
Married	968	59.79			
Unmarried	651	40.21			
**Educational level**			27.750		0.000
Primary school or below	361	22.30			
Junior high school	738	45.58			
High school and above	520	32.12			
**Family history of mental illness**			1.556		0.459
Yes	148	9.14			
No	1,471	90.86			
**Family member’s highest education**			3.984		0.136
High school or below	1,192	73.63			
College diploma or above	427	26.37			
**Family economic status**			5.562		0.062
Poor	693	42.80			
Not poor	926	57.20			
**Residential status**			42.512		0.000
Rural	575	35.52			
Urban	1,044	64.48			
**Development level of the districts**			46.755		0.000
High level	546	33.72			
Medium level	449	27.73			
Relatively low level	624	38.54			

The majority of participants were female (56.83%), married (59.79%), with less than a high school education (67.88%), not poor (57.2%) and with an urban residence (64.48%). Among them, 9.14% had a family history of mental illness and 73.63% lived in less educated families, assessed by the highest education of their family members. Independent chi-square analyses showed that the status of treatment delay may vary between/among groups with different marital status, education level, residential status and development level of the residential districts (p < 0.001).

### Trend in treatment delay over time

**Fig. 1:**
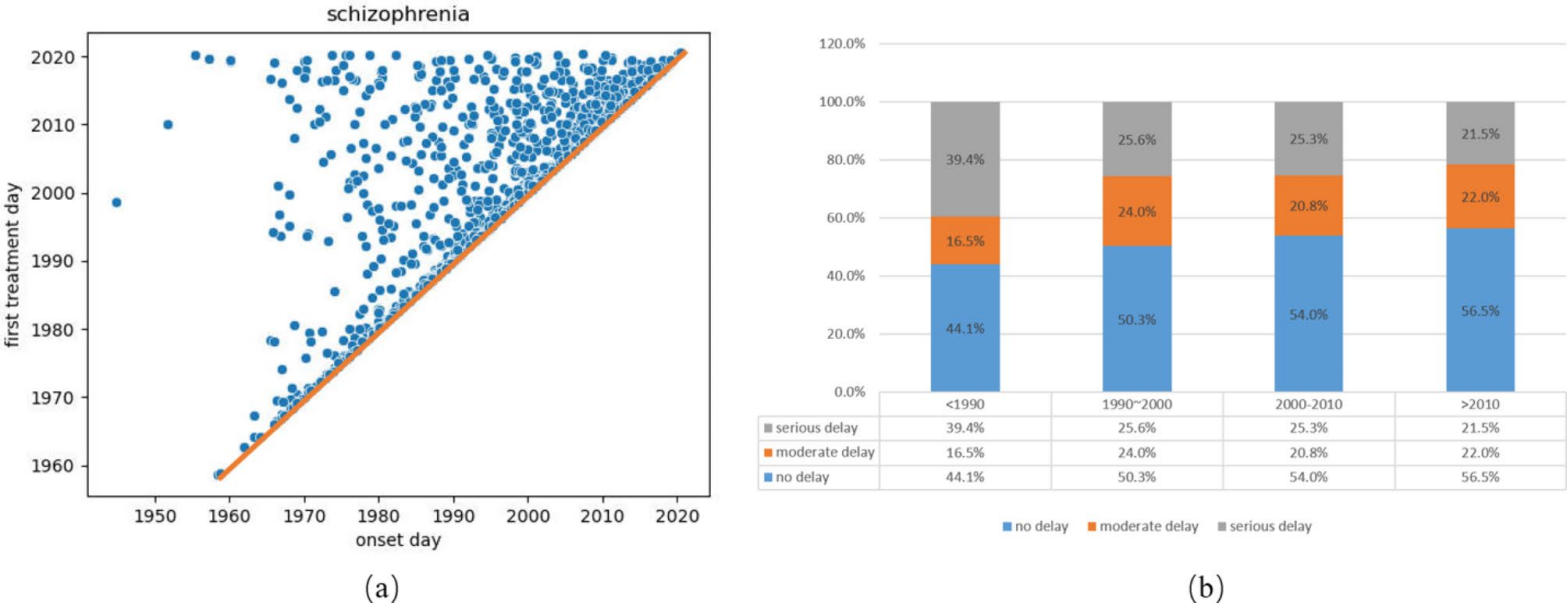
Scatter and bar charts showing trends in treatment delays over time

The development of mental health services may encourage patients to seek timely medical treatment. Figure 1 shows the trend in treatment delay for schizophrenia over time. Figure 1(a) shows the scatterplot of disease onset and treatment time. Scatter points on the red line represent patients who received antipsychotic treatment immediately after the first onset of schizophrenia. The closer to the line, the shorter the treatment delay. These scatter points were more concentrated after the 1990s, showing that patients were less likely to delay treatment in recent decades. Figure 1(b) further illustrates the delays in treatment for patients who developed schizophrenia at different time periods. Again, after the 1990s, more than half of the patients were treated within three months of onset.

### Risk factors for delayed treatment in schizophrenia

Table 2 shows the results of the ordered logistic regression, with the base being the no treatment delay group. The log likelihood ratio chi-square test (LR chi2 = 30.112, p = 0.002) indicates that the model as a whole is statistically significant. Participants’ age at onset, education level and district of residence were all found to be significant at 5% and negatively associated with treatment delay status. This suggests that early-onset patients with lower educational attainment and living in relatively well-developed areas were more likely to have delayed treatment for schizophrenia. After controlling for other variables, marital status and residential status were not significant in explaining treatment delay, although previous independence analyses suggest that treatment status may differ for these two categories.

**Table 2:** Ordered logistic regression results with base equal to no delay

Treatment delay group		Coef.	St.Err.	Z-Stat.		Prob.	95% CI		OR	
Gender (female = 0)		0.164	0.098	1.67		0.095	− 0.028, 0.356		1.178	
Age at onset		− 0.015	0.004	-3.57		0.000	− 0.023, − 0.007		0.986	
Marital status (unmarried = 0)		− 0.054	0.104	-0.52		0.603	− 0.258, 0.15		0.947	
Educational level (primary or below = 0)										
Junior high school		− 0.313	0.129	-2.43		0.005	− 0.565, − 0.06		0.731	
High school and above		− 0.386	0.147	-2.63		0.009	− 0.674, − 0.098		0.680	
Family history of mental illness (yes = 0)		0.241	0.167	1.44		0.149	− 0.087,0.569		1.273	
Family member’s highest education (high school or below = 0)		0.02	0.11	0.18		0.857	− 0.196, 0.236		1.020	
Family economic status (poor = 0)		0.161	0.105	1.53		0.126	− 0.045, 0.368		1.175	
Residential status (rural = 0)		0.074	0.119	0.63		0.531	− 0.158, 0.307		1.077	
Development level of the districts (high = 0)										
Medium level		0.136	0.126	1.08		0.013	− 0.111, 0.382		0.914	
Relatively low level		− 0.078	0.129	-0.61		0.044	− 0.33, 0.174		0.825	
cut1		− 0.266	0.242				− 0.74, 0.207			
cut2		0.631	0.242				0.156, 1.105			
Mean dependent var	0.781				SD dependent var			0.864		
Pseudo r-squared	0.009				Number of obs			1619		
Chi-square	30.112				Prob > chi2			0.002		
Akaike crit. (AIC)	3326.100				Bayesian crit. (BIC)			3396.164		

### Clinical outcomes of delayed treatment

Table 3 shows the results of binary logistic models of the clinical outcomes of treatment delay in schizophrenia. In the first model, patients who delayed treatment were less compliant with medication compared to those who received treatment on time. Medication adherence was also significantly associated with participants’ age, marital status, education level, residential status and economic status. In general, patients who were younger, married, better educated, not poor and living in urban areas had better medication adherence.

**Table 3:** Results of binary logistic models showing clinical outcomes of treatment delay

	Model-1 Medication adherence (DV: yes = 1, no = 0) OR (St.Err.)	Model-2 Comorbidity status (DV: yes = 1, no = 0) OR (St.Err.)	Model-3 Social function (DV: poor = 1, good = 0) OR (St.Err.)
Gender (female = 0)	1.088(0.123)	1.032(0.165)	1.228(0.156)
Age	0.979(0.005)***	1.032(0.007)***	1.012(0.006)
Marital status (unmarried = 0)	1.366(0.167)*	0.807(0.137)	0.661(0.091)**
Educational level (Primary school or below = 0)			
Junior high school	1.285(0.181)	0.937(0.188)	1.149(0.188)
High school and above	1.422(0.244)*	0.975(0.24 )	0.954(0.191)
Residential status (rural = 0)	1.44(0.189)**	0.79(0.152)	1.507(0.221)**
Poverty status (poor = 0)	1.766(0.209)***	0.841(0.247)*	0.642(0.196)*
Family history of mental illness (yes = 0)	1.216(0.229)	0.421(0.093)***	0.849(0.183)
Family member’s highest education (high school or below = 0)	1.244(0.162)	0.684(0.132)	0.745(0.112)
Development level of the districts (high level = 0)			
Medium level	0.932(0.142)	0.761(0.164)	2.938(0.541)***
Relatively low level	0.928(0.137)	0.873(0.182)	6.192(1.126)***
Age at onset	1.006(0.005)	0.997(0.007)	0.998(0.006)
Treatment delay (no delay = 0)			
Moderate delay	0.797(0.318)***	1.532(0.143)***	2.175(0.351)***
Severe delay	0.738(0.224)***	1.621(0.165)***	1.687(0.162)*
Medication adherence (no = 0)		0.828(0.134)	0.413(0.054)***
Comorbidity status (no = 0)			1.532(0.272)*
Constant	1.095(0.389)	0.094(0.047)***	0.072(0.031)***
**Summary of statistics**			
Pseudo R2	0.367	0.249	0.408
Prob. > chi2	0.000	0.000	0.000
Chi-square	142.915	60.078	198.360

Note: * p < 0.05; ** p < 0.01; *** p < 0.001

The second model shows a significant relationship between delayed treatment for schizophrenia and comorbidity status. Compared to the no delay group, patients who delayed treatment for their mental illness were more likely to have other chronic comorbid conditions. In addition, patients who were older, poor and had a family history of mental illness were more likely to have comorbidities.

The third model suggests that, while controlling for other variables, treatment delay had a salient relationship with patients’ poor social function. Meanwhile, marital status, residential status, district of residence, medication adherence and comorbidity status were also related to patients’ social function, as those patients who were unmarried, poor, lived in urban but less developed districts, were medication non-adherence and had comorbidities were more likely to have poor social function.

### Potential impact of delayed treatment on families

Table 4 examines the outcomes for schizophrenia at the household level, using treatment delay and its clinical outcomes as explanatory variables. It suggests that, after controlling for other variables, treatment delay can still have a wide range of negative effects on families. In particular, compared with the no-delay group, severe treatment delay in schizophrenia may have more severe effects on the family economy, daily life, social relationships and the health of other family members (p < 0.05). In addition, treatment delay in schizophrenia may also affect families by affecting patients’ medication adherence, comorbidity status and social functioning. As shown in Table 4, medication adherence is related to perceived burden in daily life and social relationships, while comorbidity and poor social functioning are related to all four dimensions of family burden.

**Table 4:** Results of the linear regression models showing family outcomes of treatment delay

	Model-1 Economic burden B (St.Err.)	Model-2 Daily life burden B (St.Err.)	Model-3 Relationship burden B (St.Err.)	Model-4 Health burden B (St.Err.)
Gender (female = 0)	0.035(0.034)	0.025(0.036)	0.03(0.039)	0.059(0.04)
Age	− 0.004(0.002)**	− 0.002(0.002)	− 0.001(0.002)	− 0.002(0.002)
Marital status (unmarried = 0)	− 0.037(0.038)	− 0.069(0.039)	− 0.058(0.043)	− 0.078(0.044)
Educational level (primary school or below = 0)				
Junior high school	0.118(0.045)***	0.124(0.047)***	0.141(0.051)***	0.128(0.052)**
High school and above	0.177(0.053)***	0.183(0.055)***	0.177(0.06 )***	0.187(0.061)***
Residential status (rural = 0)	− 0.119(0.041)***	− 0.051(0.043)	− 0.041(0.047)	− 0.009(0.048)
Family history of mental illness (yes = 0)	0.024(0.058)	0.023(0.061)	− 0.025(0.066)	− 0.006(0.068)
Family member’s highest education (high school or below = 0)	− 0.103(0.039)	− 0.082(0.041)	− 0.08(0.044)	− 0.095(0.045)
Development level of the districts (high level = 0)				
Medium level	− 0.123(0.046)***	− 0.118(0.048)**	− 0.156(0.052)***	− 0.184(0.053)***
Relatively low level	− 0.049(0.045)*	− 0.108(0.047)**	− 0.17(0.052)***	− 0.161(0.053)***
Age at onset	− 0.002(0.002)	− 0.002(0.002)	− 0.001(0.002)	− 0.002(0.002)
Treatment delay (no delay = 0)				
Moderate delay	0.066(0.045)*	0.019(0.047)	0.064(0.051)	0.03(0.052)
Severe delay	0.037(0.039)**	0.003(0.041)*	0.054(0.045)**	0.082(0.046)*
Medication adherence (no = 0)	0.008(0.036)	− 0.064(0.038)*	− 0.054(0.041)**	− 0.046(0.042)
Comorbidity status (no = 0)	0.061(0.051)**	0.122(0.053)**	0.164(0.058)***	0.126(0.059)**
Patient’s social function (good = 0)	0.025(0.04)*	0.019(0.042)*	0.067(0.046)**	0.08(0.047)*
Constant	2.745(0.111)***	2.586(0.116)***	2.508(0.126)***	2.44(0.129)***
**Summary of statistics**				
R2	0.142	0.134	0.138	0.146

Note: * p < 0.05; ** p < 0.01; *** p < 0.001

In addition to factors related to treatment delay, the perceived burden of schizophrenia was also associated with the patients’ age, level of education, residential status and region of residence. In particular, families with a relatively well-educated patient and living in more affluent areas may perceive a greater burden of schizophrenia in all four dimensions. The perceived economic burden is higher for families with a younger patient and living in rural areas.

## Discussion

Currently, there are a number of effective treatment options for patients with schizophrenia, and about one third can make a full recovery if they are treated appropriately and in a timely manner [24]. However, people with schizophrenia often delay treatment for reasons and outcomes that are not fully understood. Although the treatment and management of patients with schizophrenia has been listed as an important public health task [25], literature on the prevalence, risk factors, and multiple outcomes of treatment delay in schizophrenia is still lacking in China. In this study, a representative sample dataset from a metropolitan context was used to address this knowledge gap.

In this study, the median treatment delay for schizophrenia is 89 days (approximately 13 weeks), which is similar to the conclusions drawn from other domestic studies [20, 26]. It is shorter than studies based in America [27], Saudi Arabia [16] and South America [28], but similar to Canada (14.6 weeks) [29]. These comparisons are indirect, and further research is needed to evaluate the comparisons. In China, more policy attention has been paid to reduce the treatment gap for mental illness [25, 30], advances in mental health services have enabled patients to seek timely treatment. When comparing delays in different time periods, this study finds a notable reduction in serious treatment delays in recent years.

Similar to other studies [15, 16], this study found that early onset is associated with treatment delay in schizophrenia. In societies where people have limited knowledge about the illness, early onset is more likely to be perceived as defiant behavior in adolescence [31]. Patients’ level of education was also negatively associated with treatment delay, which is consistent with other studies [32, 33]. Compared to patients with primary education, those with higher education were more likely to seek timely treatment. One explanation could be that mental health issues were poorly represented in primary education curricula, so people were less aware of them [34]. It could also be explained the other way round that people who delayed treatment for schizophrenia had worse clinical outcomes, which hindered them from getting better education [17]. This study also found that patients living in developed districts were more likely to delay treatment than their counterparts. This is unlikely to be due to the accessibility of mental health services. More research is needed to find out why.

Previous studies have shown that better educated families would facilitate earlier access to treatment [8, 35]. However, this study did not find a salient relationship between the highest level of education of family members and treatment delay for schizophrenia. To achieve the goal of effective early intervention, the public may need more specific knowledge about mental illness [20]. Familiarity with the illness should also have an impact on treatment delay. Family members with a history of mental illness were found to be less sensitive to non-specific prodromal symptoms but more sensitive to those positive symptoms, compared to those without a family history [36]. However, this study did not found a salient relationship between family history of mental illness and treatment delay in schizophrenia, which is consistent with some other studies [37]. The cultural beliefs associated with seeking treatment for schizophrenia deserve further investigation.

Although the negative impact of treatment delay has been widely recognized, the extent and nature of its impact has varied [17, 38]. In this study, we found that treatment delay in schizophrenia has significant multiple effects on patients and families. At an individual level, it was significantly associated with patients’ medication adherence, comorbidity status and social functioning ratings, consistent with other studies [35, 39]. These negative clinical effects may spill over to the household level, as they were all associated with different facets of family burden. Meanwhile, after controlling for these factors, the association between treatment delay in schizophrenia and family burden remained significant. Further in-depth qualitative studies should be considered to uncover more mechanisms by which treatment delay may exert impacts on families, particularly on long-term family development.

This study has some limitations. First, the definition of treatment delay as the length of time between the first onset of schizophrenia and the start of psychiatric treatment may have some potential biases that need to be considered. On one hand, the ‘first onset’ data were based on hospital records obtained by asking patients when they were admitted to hospital. There may be problems with inaccurate recall, which means that they may have misremembered the time of onset. On the other hand, only patients with hospital records were recruited for the survey. Those who never went to hospital but chose other forms of treatment cannot be identified. Second, factors such as internalized stigma, social support and family members’ perceptions of the illness may also contribute to explaining treatment delay in schizophrenia [6, 8, 20]. Due to the use of secondary data, these variables not included in the questionnaire cannot be tested. Third, treatment delay is a highly contextual issue. This study was rooted in the metropolitan context of China, where mental health resources are relatively adequate, and the findings may not be fully applicable to other contexts. Fourth, the sample in this study had a high average age (mean: 55.44), with those aged over 55 years accounting for 51.27% of the total sample. Therefore, this study may be limited in fully representing the treatment status of younger people. Further research is needed to understand the issues of treatment delay in younger people.

In this study, treatment delay in schizophrenia is significantly associated with early onset and low educational attainment, suggesting that targeted interventions to increase mental health literacy in the early education phase may be critical to reduce treatment delay. Families can play an important role in shaping patients’ treatment behaviors [36]. However, this study did not find a salient relationship between family characteristics (i.e. economic status, highest educational level of family member and family history of mental illness) and treatment delay. Family members may not believe that patients are ill, or they may lack confidence in treatment and recovery, even among those with higher levels of education [20]. Mental health education for the general public should be considered to reduce the potential negative effects of treatment delay in schizophrenia.

## Conclusion

This study enriches the empirical evidence on the prevalence of treatment delay in schizophrenia, its associated factors and multiple outcomes in a metropolitan context in China. It contributes to informing further targeted policies in China, as well as to the body of evidence on treatment delay in severe mental illness in low-and middle-income countries. It suggests that treatment delay remains a major challenge, even in the metropolis where mental health services are relatively adequate, especially for people with earlier onset, lower educational attainment and living in relatively well-developed areas. Reducing the treatment gap for schizophrenia is not only about increasing the supply of mental health services, but also about getting people to use them in time. Intervention and education efforts are urgently needed to reduce treatment delay in schizophrenia. Failure to do so can result in poor clinical outcomes and a significant negative impact on families.

## Data Availability

The datasets used and analyzed in this study are available from the corresponding author on reasonable request.

## References

[1] Saparia P (2022). Schizophrenia: a systematic review. J Clin Experimental Psychol.

[2] Olfson M (2015). Premature mortality among adults with schizophrenia in the United States. JAMA Psychiatry.

[3] Yu Y-H, Peng M-M (2022). Development and Poverty Dynamics in severe Mental illness: a modified Capability Approach in the chinese context. Int J Environ Res Public Health.

[4] Albert N (2017). The effect of duration of untreated psychosis and treatment delay on the outcomes of prolonged early intervention in psychotic disorders. NPJ Schizophrenia.

[5] Cohen CI, Meesters PD, Zhao J (2015). New perspectives on schizophrenia in later life: implications for treatment, policy, and research. The Lancet Psychiatry.

[6] Stagnaro JC (2019). Delays in making initial treatment contact after the first onset of mental health disorders in the Argentinean Study of Mental Health Epidemiology. Epidemiol Psychiatric Sci.

[7] Dutta M (2019). Factors responsible for delay in treatment seeking in patients with psychosis: a qualitative study. Indian J Psychiatry.

[8] Franz L (2010). Stigma and treatment delay in first-episode psychosis: a grounded theory study. Early Interv Psychiat.

[9] Chen H (2020). Time delay in seeking treatment for first-episode schizophrenia: a retrospective study. Early Interv Psychiat.

[10] Hasan AA, Musleh M (2017). Barriers to seeking early psychiatric treatment amongst first-episode psychosis patients: a qualitative study. Issues Ment Health Nurs.

[11] Lilford P, Rajapakshe OBW, Singh SP (2020). A systematic review of care pathways for psychosis in low-and middle-income countries. Asian J Psychiatry.

[12] Wong DFK, Li JCM (2014). Cultural influence on Shanghai chinese people’s help-seeking for mental health problems: implications for social work practice. Br J Social Work.

[13] Bhui K, Ullrich S, Coid JW (2014). Which pathways to psychiatric care lead to earlier treatment and a shorter duration of first-episode psychosis?. BMC Psychiatry.

[14] Basu S (2015). Does ethnicity have an impact on duration of untreated psychoses: a retrospective study in Singapore. Int J Soc Psychiatry.

[15] Hui CLM (2015). Clinical and social correlates of duration of untreated psychosis among adult-onset psychosis in H ong K ong C hinese: the JCEP study. Early Interv Psychiat.

[16] Al Fayez H (2017). Duration of untreated psychosis and pathway to care in R iyadh, S audi a rabia. Early Interv Psychiat.

[17] Cross SP (2018). Variability in clinical outcomes for youths treated for subthreshold severe mental disorders at an early intervention service. Psychiatric Serv.

[18] Reichert A, Jacobs RJHe. *The impact of waiting time on patient outcomes: evidence from early intervention in psychosis services in E ngland*. 2018. 27(11): p. 1772–87. 10.1002/hec.3800 PMC6221005 30014544

[19] Huang Y et al. *Prevalence of mental disorders in China: a cross-sectional epidemiological study* 2019. 6(3): p. 211–224. 10.1016/S2215-0366(18)30511-X 30792114

[20] Qiu Y (2019). Factors related to duration of untreated psychosis of first episode schizophrenia spectrum disorder. Early Interv Psychiat.

[21] Stone WS (2020). Association between the duration of untreated psychosis and selective cognitive performance in community-dwelling individuals with chronic untreated schizophrenia in rural China. JAMA Psychiatry.

[22] Ran M-S et al. *Duration of untreated psychosis (DUP) and outcome of people with schizophrenia in rural China: 14-year follow-up study*. 2018. 267: p. 340–5. 10.1016/j.psychres.2018.06.043 29957551

[23] Karson C et al. *Long-term outcomes of antipsychotic treatment in patients with first-episode schizophrenia: a systematic review*. Neuropsychiatr Dis Treat, 2016: p. 57–67. 10.2147/NDT.S96392 PMC4708960 26792993

[24] Vita A, Barlati S (2018). Recovery from schizophrenia: is it possible?. Curr Opin Psychiatry.

[25] Liang D, Mays VM, Hwang W-C (2018). Integrated mental health services in China: challenges and planning for the future. Health Policy Plann.

[26] Wang J (2009). Duration of untreated illness and its influential factors in schizophrenia. Shanghai Archives of Psychiatry.

[27] Correll CU (2014). Cardiometabolic risk in patients with first-episode schizophrenia spectrum disorders: baseline results from the RAISE-ETP study. JAMA Psychiatry.

[28] González-Valderrama A (2017). Duration of untreated psychosis and acute remission of negative symptoms in a S outh a merican first‐episode psychosis cohort. Early Interv Psychiat.

[29] Lyne J (2017). Duration of active psychosis and first-episode psychosis negative symptoms. Early Interv Psychiat.

[30] Que J, Lu L, Shi L. *Development and challenges of mental health in China*. Gen Psychiatry, 2019. 32(1). 10.1136/gpsych-2019-100053 PMC6551437 31179426

[31] De Girolamo G (2012). Age of onset of mental disorders and use of mental health services: needs, opportunities and obstacles. Epidemiol Psychiatric Sci.

[32] Seabury SA (2019). Measuring the lifetime costs of serious mental illness and the mitigating effects of educational attainment. Health Aff.

[33] Nishio A (2018). Factors that influence delaying initial psychiatric treatment in rural Cambodia: a pilot study. PLoS ONE.

[34] Thirthalli J (2017). Rural–urban differences in accessing mental health treatment in patients with psychosis. Int J Soc Psychiatry.

[35] Dixon LB, Holoshitz Y, Nossel I (2016). Treatment engagement of individuals experiencing mental illness: review and update. World Psychiatry.

[36] Lutgens D (2015). The impact of caregiver familiarity with mental disorders on timing of intervention in first-episode psychosis. Early Interv Psychiat.

[37] Norman RM, Malla AK, Manchanda R (2007). Delay in treatment for psychosis. Soc Psychiatry Psychiatr Epidemiol.

[38] Lally J (2017). Remission and recovery from first-episode psychosis in adults: systematic review and meta-analysis of long-term outcome studies. Br J Psychiatry.

[39] Korczak DJ, Goldstein BI (2009). Childhood onset major depressive disorder: course of illness and psychiatric comorbidity in a community sample. J Pediatr.

